# Increased feeding frequency prior to farrowing: effects on sow performance

**DOI:** 10.1093/tas/txac062

**Published:** 2022-05-16

**Authors:** Bryony S Tucker, Kiro R Petrovski, Jessica R Craig, Rebecca S Morrison, Robert J Smits, Roy N Kirkwood

**Affiliations:** School of Animal and Veterinary Sciences, The University of Adelaide, Roseworthy, SA 5371, Australia; School of Animal and Veterinary Sciences, The University of Adelaide, Roseworthy, SA 5371, Australia; Davies Livestock Research Centre, The University of Adelaide, Roseworthy, SA 5371, Australia; Research and Innovation, Rivalea Australia Pty Ltd, Corowa, NSW 2646, Australia; Research and Innovation, Rivalea Australia Pty Ltd, Corowa, NSW 2646, Australia; Research and Innovation, Australian Pork Limited, Barton, ACT 2600, Australia; School of Animal and Veterinary Sciences, The University of Adelaide, Roseworthy, SA 5371, Australia

**Keywords:** farrowing duration, feeding frequency, sow, stillborn

## Abstract

Reducing the interval between the consumption of the last meal and the start of farrowing is suggested to increase the energy available to sows during farrowing, potentially reducing the farrowing duration and easing piglet births. The present study aimed to examine whether increasing feeding frequency from one to two feeds within standard production hours (0700 to 1500 hours) would produce a difference in farrowing duration and/or stillborn numbers. From entry to farrowing crates (110 ± 1 d gestation) to farrowing (116 ± 1 d gestation), multiparous sows (*n* = 118) were fed a daily fixed amount of feed either once at 0800 hours or in two meals at 0800 and 1300 hours. Sow weights and backfat depths were recorded on entry and exit from the farrowing crate. Litter size and weight were recorded 24 h after farrowing and on day 21 of lactation. Sows fed twice had a shorter farrowing duration and fewer stillborn piglets than those fed once (2.21 ± 0.56 h vs. 3.25 ± 0.52 h; *P* = 0.001). The interaction between treatment and farrowing duration showed that sows fed twice have a reduced farrowing duration and had significantly lower stillborn rates than those fed once or those fed twice with longer farrowing durations (*P* < 0.001). These findings suggest that increasing feeding frequency prior to farrow can reduce the farrowing duration and stillborn numbers in some sows, however, some sows remain with a high stillborn rate regardless of feeding frequency. Piglet average daily gain was greater in once-fed sows, but fewer of these sows remained in the herd at subsequent farrowing. Further, subsequent total born and born alive were higher in twice-fed sows. Feeding sows at a higher frequency can improve farrowing performance in some sows and could increase the longevity of the sow in the herd.

## INTRODUCTION

Selection for larger litter sizes has resulted in a renewed focus on management strategies to prepare the sow for a faster farrowing to optimize born alive and piglet preweaning survival. A common problem associated with larger litters is a longer farrowing duration, which may result from an energy deficit in the sow and/or complications during the expulsion process (Manu et al., 2017, 2019). The relationship between a prolonged farrowing duration and increase stillbirth rate or reduce neonatal viability are well known (van Dijk et al., 2005; Oliviero et al., 2010; Langendijk et al., 2018). Arguably, the stillbirth of piglets is one of the largest contributors to reduced litter size weaned and thus reduced production returns.

Gestation and lactation feeding strategies have been the focus of much nutritional research and management decisions (Campos et al., 2012). The transition period from gestation to lactation (7 d before to 7 d after farrowing) has recently been highlighted as an important phase for determining farrowing performance (Theil et al., 2011; Langendijk and Fleuren, 2018; Pedersen et al., 2020; Feyera et al., 2021). Increasing feeding frequency to transition sows resulted in a reduced time from last feed to farrowing and, in those sows having feed-to-farrow intervals less than 3.1 h, also higher arterial blood glucose concentrations and reduced farrowing durations (Feyera et al., 2018). In contrast, Gourley et al. (2020) found no reduction in farrowing duration or stillborn rate with increased prepartum feeding frequency. It is likely that geographic location, environmental conditions, genetics, and other management practices may have impacted the results of these studies, thus questioning how effective reducing time from feeding to farrowing is across different farms. Further, the difference between the two studies was suggested by Gourley et al. (2020) to be due to the sows of Feyera et al. (2018) having a higher mean farrowing duration and total born in their study population.

Gourley et al. (2020) fed their sows at 0100, 0700, 1300, and 1900 hours. However, most farms are not set up or staffed to deliver feed at these times, especially when sows are required to be hand-fed. Therefore, our study was designed to test two feeding frequencies within a standard commercial production system with full staffing between the hours of 0700 and 1500 hours to determine whether a difference in farrowing duration and/or stillborn numbers occurred and, thus, be useful for farming practices. We hypothesized that increasing feeding frequency in the transition period before farrowing would reduce the farrowing duration and the number of piglets stillborn.

## MATERIALS AND METHODS

This experiment was conducted under commercial conditions at Corowa, NSW, Australia, from August to September. The experiment was approved by the Rivalea Pty Ltd. Animal Care and Ethics Committee (Protocol 19B014) in accordance with the Australian Code for the Care and Use of Animals for Scientific Purposes (National Health and Medical Research Council, 2013).

### Animals and Experimental Design

At entry to the farrowing house, 118 mixed parity (1 to 8) Large White × Landrace sows were allocated to dietary treatment of either one feed at 0800 hours or two feeds at 0800 and 1300 hours, given each day until farrowing commenced. All feeds were provided before farrowing via hand-feeding by one of two trained stock people. Sows were allocated to their treatment based on P2 backfat depth and parity at entry to the farrowing house, balanced across treatment groups. A daily allocation of 3.8 kg of a commercial lactation feed (Table 1) was provided to both treatment groups (as one feed or split as two 1.9 kg feeds).

**Table 1.** Composition of lactation diet

Item	% of fed basis
Wheat	57.00
Barley	10.00
Mill mix	6.70
Canola meal, 38% CP	10.00
Meat meal, 58% CP	3.33
Soybean meal, 46%	2.5
Fish oil	0.40
Tallow	6.00
Betaine	0.40
Limestone	1.00
Magnesium sulfate	0.4
Potassium chloride	0.2
Lysine micro	0.43
Threonine micro	0.13
Tryptophan micro	0.04
Sow replace pak micro	0.2
Repro blend micro	0.05
Vitamin and mineral premix	0.47
Enzymes^1^	0.02
Antioxidant^2^	0.04
Phytase^3^	0.01
Yeast product^4^	0.01
Insoluble fibre^5^	0.67
Calculated composition:	
DE, MJ/kg	14.93
Crude protein, %	16.04
Crude fat, %	7.40
Crude fiber, %	4.07
Ash, %	5.36
Available SID lysine, %	1.01
Calcium, %	0.90
Phosphorus, %	0.98

Commercial product Rovabio Max (Adisseo, Antony, France).

Commercial product Endox Dry (Kemin Industries, Des Moines, Iowa, USA).

Commercial product Quantum Blue (AB Vista, Marlborough, UK).

Commercial products Proternative 10 Titan (Lallemand Animal Nutrition, Australia).

Arbocel (Rettenmaier & Söhne, Rosenberg, Germany).

For all sows that were farrowed, the number of piglets born alive and stillborn were recorded. A subset of sows (*n* = 33) who completed farrowing during extended staffed hours of 0700 and 2000 hours were recorded for farrowing duration measured from birth of first and last piglets. The day following completion of farrowing, gestation length, litter size, and litter weight (of live piglets) were recorded for each sow. Minimal fostering was permitted to standardize litter size to available functional teat number and the new litter size was recorded. Litter weight and litter size were recorded on day 21 of lactation for all litters, just prior to weaning. All sows were weighed and backfat depths were recorded at weaning.

### Housing and Management

Sows were moved into the farrowing house at day 110 (± 2 d) of gestation and their litters weaned at day 21 (± 2 d) of lactation. Prior to entry, sows were weighed and backfat depth at the P2 position was obtained using an ultrasound machine and probe. Sows were individually housed in traditional slatted floor farrowing crates, each having a solid floored creep area with an infrared heat lamp for the piglets. Sows were monitored daily for general health and welfare throughout the farrowing house period. For this study, upon their due date, sows were monitored for 13 h daily for the onset of farrowing or farrowing difficulty. All sows are farrowed naturally without exogenous hormonal induction. Farrowing assistance was provided if the sow showed signs of distress during farrowing and/or if 45 min had elapsed from the birth of the last piglet with no farrowing progress evident (Cowart, 2007). A piglet was considered live-born if movement and/or breathing were detected following expulsion. During lactation, sows were fed to appetite the lactation diet as per standard production lactation management on this farm. After weaning, sows returned to the normal production system and subsequent reproductive data were collected from electronic farm data records.

### Statistical Analysis

All statistical analyses were performed using SAS version 9.4 (Statistical Analysis Software, Cary, NC, USA). The data were analyzed as two data sets: subset (observed sows) = 33 sows observed to be farrowing during staffed hours; main data set = 99 sows provided treatment within the 2 experimental sheds. For the statistical analyses, all data sets were bootstrapped at a root of 8 using PROC SURVEYSELECT subset = 164 sow observations and main data set = 509 sow observations for the analyses. The accuracy of the data was tested using PROC MEANS with all means of the bootstrapped data being similar to the original data to at least the second decimal point. Prior to the analyses, some data were manipulated:

- Farrowing durations were allocated to two categories based on results by Feyera et al. (2018)—farrowing duration ≤3.47: FD 1; farrowing duration >3.47: FD 2.
- Total born was categorized into three levels—ls 1: 7 to 9 piglets; ls 2: 10 to 14 piglets; and ls 3: ≥15 piglets.
- P2 backfat change was categorized into three groupings—BF 1: <−6 mm; BF 2: −6 to −0.1 mm; BF 3: ≥0.1.
- Sow weight at entry and change during lactation were corrected for an estimated weight of conceptus using the equation by NRC (2012).

$$\begin{array}{ll} & Weight\;of\;conceptus=\\ & \frac{\left[exp\left(8.621-21.02\;\times exp\left(-0.053\;\times gestation,\;d\right)+0.114\;\times total\;\;\;born,\;n\right)\right]}{1,000}, \end{array}$$

- Weight change was categorized into four groupings—Wt 1: <−13.68 kg; Wt 2: −13.68 to −6.07 kg; Wt 3: −6.08 to 2.55 kg; and Wt 4: >2.55 kg.
- Piglet birth weights were categorized into four groups—pbw 1: <1.41 kg; pbw 2: 1.41 to 1.59 kg; pbw 3: 1.59 to 1.76 kg; pbw 4: >1.76 kg.

The effect of treatment, litter size, days from entry to farrow, and parity on farrowing duration was estimated using a mixed model in PROC MIXED for the subset, as presented in equation 1:


$$\begin{array}{l} Farrowing\;duration=treatment{\;\;\;}_{p2,s}+litter\;size_{p2,s}\\ +\;\;days\;from\;entry\;to\;farrow_{p2,s}+\;parity_{p2,s}, \end{array}$$ (1)

where p2 is the p2 backfat at entry; s is the room. The preliminary model also tested the effects of the previous total born but it was found to be not significant (*P* > 0.1) and so excluded from the final model. The outputs of the model were least-square means, their respective standard errors, and the difference between least-square means. The level of significance was set at *P* < 0.05.

The effect of treatment, farrowing duration, litter size, days from entry to farrow, and parity on stillborn number was estimated using a mixed model in PROC MIXED for subset and the main data set (where variables are applicable), as presented in equation 2:


$$\begin{array}{l} \begin{array}{ll} & Stillborn=treatment_{p2,s}+farrowing\;duration_{p2,s}\\ & +litter\;size_{p2,s}+\;days\;from\;entry\;to\;farrow_{p2,s}\\ & +\;parity_{p2,s}\;+\;{\left(treatment\times farrowing\;duration\right)}_{p2,s}, \end{array} \end{array}$$ (2)

where p2 is the p2 backfat at entry; s is the room. The preliminary model also tested the effects of the previous total born but it was found to be not significant (*P* > 0.1) and was excluded from the final model. The outputs of the model were least-square means, their respective standard errors, and the difference between least-square means. The level of significance was set at *P* < 0.05.

The effect of treatment, sow P2 backfat, and piglet birth weight category on average piglet weight gain were estimated using a mixed model in PROC MIXED for the main data set, as presented in equation 3:


$$\begin{array}{ll} & Average\;piglet\;gain/day=treatment_{d,l,p,p2,s}\\ & +piglet\;\;\;birth\;\;\;weight\;\;\;category_{d,l,p,p2,s}, \end{array}$$ (3)

where d is the days from entry to farrow, l is the litter size, p is the parity, p2 is the sow backfat at entry, and s is the room. The preliminary model also tested the effect of previous litter size but was found to be not significant (*P* > 0.1) and so excluded from the final model. The outputs of the model were least-square means, their respective standard errors, and the difference between least-square means. The level of significance was set at *P* < 0.05.

The effect of treatment, litter size, and weight change on P2 backfat change and weight change were estimated using a mixed model in PROC MIXED for the main data as presented in equations 4 and 5:


$$\begin{array}{ll} P2\;backfat\;change=\; & treatment_{ap,p,\;p2,s}+litter\;size_{ap,p,\;p2,s}\\ & +weight\;change_{ap,p,\;\;\;p2,s}, \end{array}$$ (4)

$$\begin{array}{ll} Weight\;change= & \;treatment_{ap,,p,\;p2,s}+litter\;size_{ap,p,\;\;\;p2,s}\\ & +P2\;backfat\;change_{ap,,p,\;\;\;p2,s}, \end{array}$$ (5)

where ap is the average piglet weight change, p is the parity, p2 is the p2 backfat at entry, and s is the room. The preliminary model also tested the effects of the previous total born, days from entry to farrow, and farrowing duration, but these were found to be not significant (*P* > 0.1) and were excluded from the final model. The outputs of the model were least-square means, their respective standard errors, and the difference between least-square means. The level of significance was set at *P* < 0.05.

The effects of treatment, days from entry to farrow, litter size, average piglet weight change, parity, and weight change on subsequent wean to estrus period, total born, and born alive were estimated using a mixed model in PROC MIXED for the main data set, as seen in equations 6–8.


$$\begin{array}{l} \begin{array}{ll} & Wean\;to\;oestrus=treatment_{fd,s}+days\;from\;entry\;to\;farrow_{fd,s}\\ & +\;litter\;size_{fd,s}+average\;piglet\;weight\;change_{fd,s}\\ & +weight\;change_{fd,s}+parity_{fd,s}, \end{array} \end{array}$$ (6)

$$\begin{array}{l} \begin{array}{ll} & Subsequent\;total\;born=treatment_{fd,s}+days\;from\;entry\;to\;farrow_{fd,s}\\ & +litter\;\;\;size_{fd,s}+average\;piglet\;weight\;change_{fd,s}\\ & +weight\;change_{fd,s}+parity_{fd,s}, \end{array} \end{array}$$ (7)

$$\begin{array}{l} \begin{array}{ll} & Subsequent\;born\;alive=treatment_{fd,s}+days\;from\;entry\;to\;farrow_{fd,s}\\ & +litter\;size_{fd,s}+average\;piglet\;weight\;change_{fd,s}\\ & +weight\;change_{fd,s}+parity_{fd,s}, \end{array} \end{array}$$ (8)

where fd is the farrowing duration, s is the room. The preliminary model also tested the effects of the previous total born but it was found to be not significant (*P* > 0.1) and so excluded from the final model. The outputs of the model were least-square means, their respective standard errors, and the difference between least-square means. The level of significance was set at *P* < 0.05.

Chi-squared test in PROC FREQ was used to determine the number of sows removed from each treatment group in subsequent mating.

## RESULTS

A summary of data set raw mean values are presented in Table 2.

**Table 2.** Mean ± standard deviation for raw dataset

	N	Mean
Parity	118	3.3 ± 1.9
Days from entry to farrow	118	8.7 ± 1.4
P2 entry, mm	113	21.2 ± 5.3
P2 backfat exit, mm	112	17.3 ± 4.8
P2 backfat change, mm	110	−3.9 ± 3.0
Weight entry, kg	112	262.6 ± 25.3
Weight exit, kg	112	256.0 ± 28.5
Weight change, kg	112	−6.06 ± 13.7
Average piglet weight change, kg	113	1.5 ± 0.4
Stillborn, %	118	7.4 ± 7.4
Born alive, %	118	92.6 ± 18.1
Litter size (born alive + stillborn)	118	14.9 ± 2.7
Farrowing duration, h	33	4.02 ± 1.8
24 h Litter size	118	12.9 ± 2.1

24 h litter size is the post fostering litter size.

### Farrowing Data

**Figure 1.**
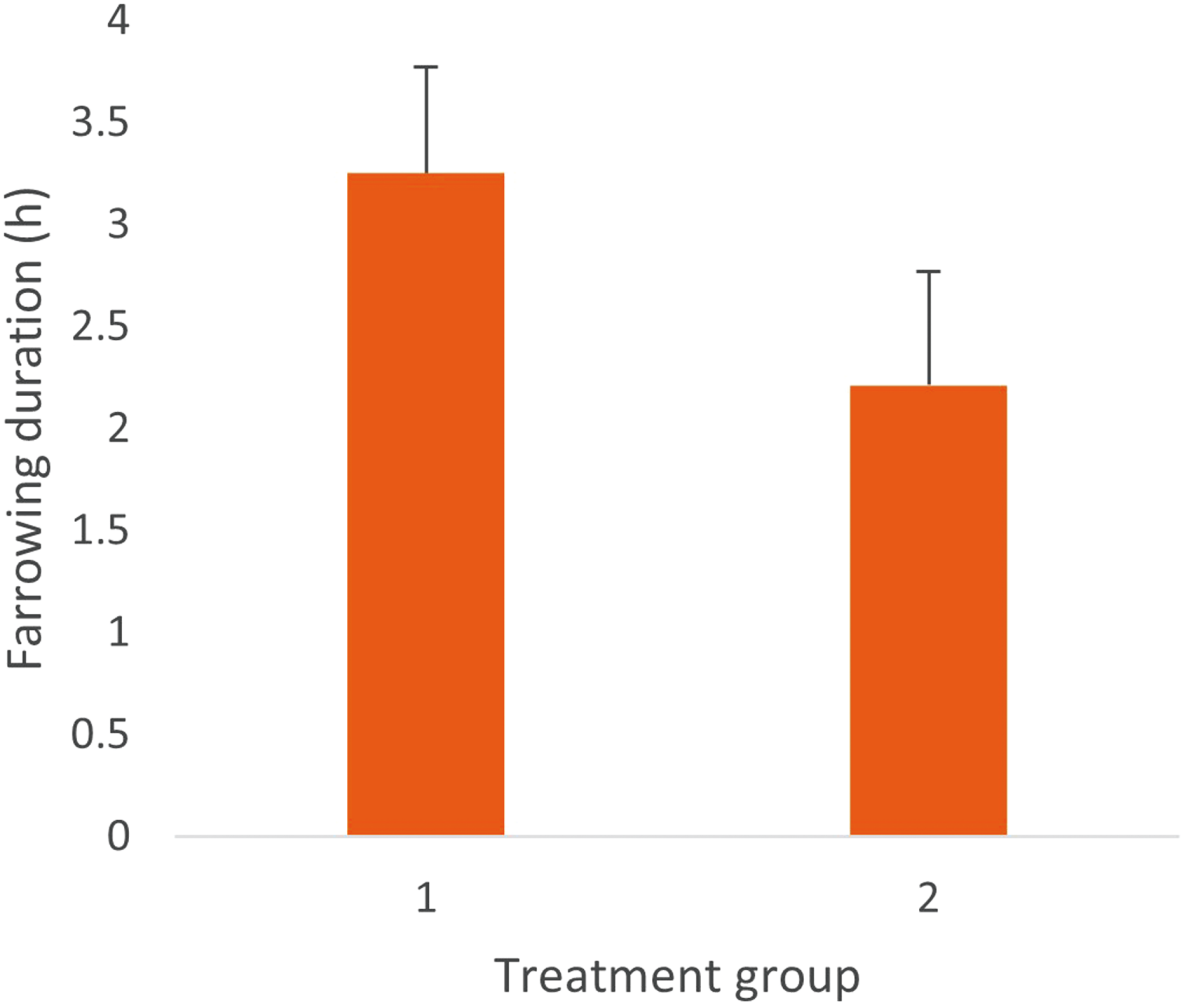
Means ± standard error of farrowing duration by treatment group for 33 sows bootstrapped at a root of 8 (164 sow observations). Treatment 1 = one feed a day. Treatment 2 = two feeds a day. Accounting for sow backfat at entry and room in the model.

Farrowing duration was reduced with increased feeding frequency (*P* < 0.001; Figure 1). Further, as litter size increased so did farrowing duration (*P* < 0.001; ls1 = 1.6 ± 0.86 h; ls2 = 2.8 ± 0.51 h; ls3 = 3.7 ± 0.44 h). As days from entry to farrowing increased from 6 to 9 d so did farrowing duration but, at 10 d, farrowing duration decreased (9 d 4.58 ± 0.7 h vs. 10 d 2.53 ± 0.36 h; *P* < 0.001).

**Figure 2.**
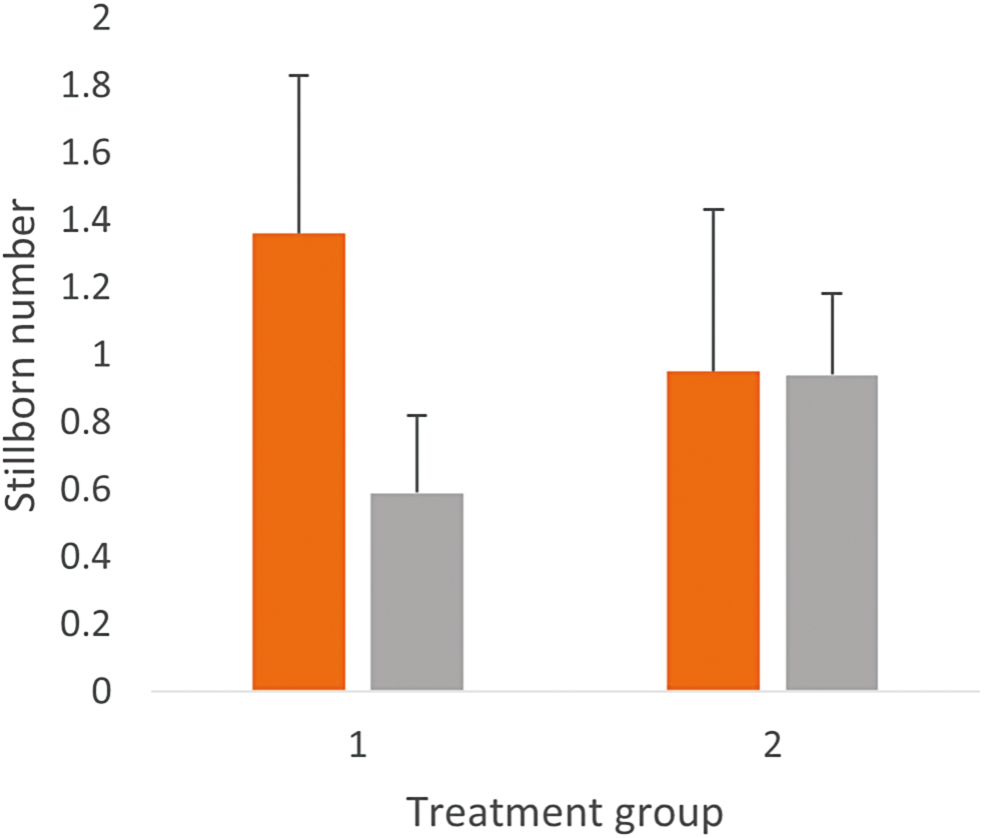
Mean ± standard error of stillborn number by treatment group two data sets bootstrapped at a root of 8; orange = 33 sows whose farrowings were observed (164 sow observations) and grey = 118 sows on trial (509 sow observations). Treatment 1 = one feed a day. Treatment 2 = two feeds a day. Accounting for sow backfat and room in the model.

Increased feeding frequency was associated with decreased stillborn in the subset of sows where farrowing duration was observed (*P =* 0.004; Figure 2). However, in the main dataset, the reverse was true (*P* < 0.001). In both data sets, sows who spent 6 d from entry to farrow had higher stillborn numbers (subset = 2.68 ± 0.57 and the main data = 1.01 ± 0.33) than sows who spent longer prior to farrowing (subset 8 d, 0.59 ± 0.47; main data 8 d, 0.50 ± 0.23; *P* < 0.001). Sows with smaller litter sizes had lower stillbirth rates (*P* < 0.001).

**Figure 3.**
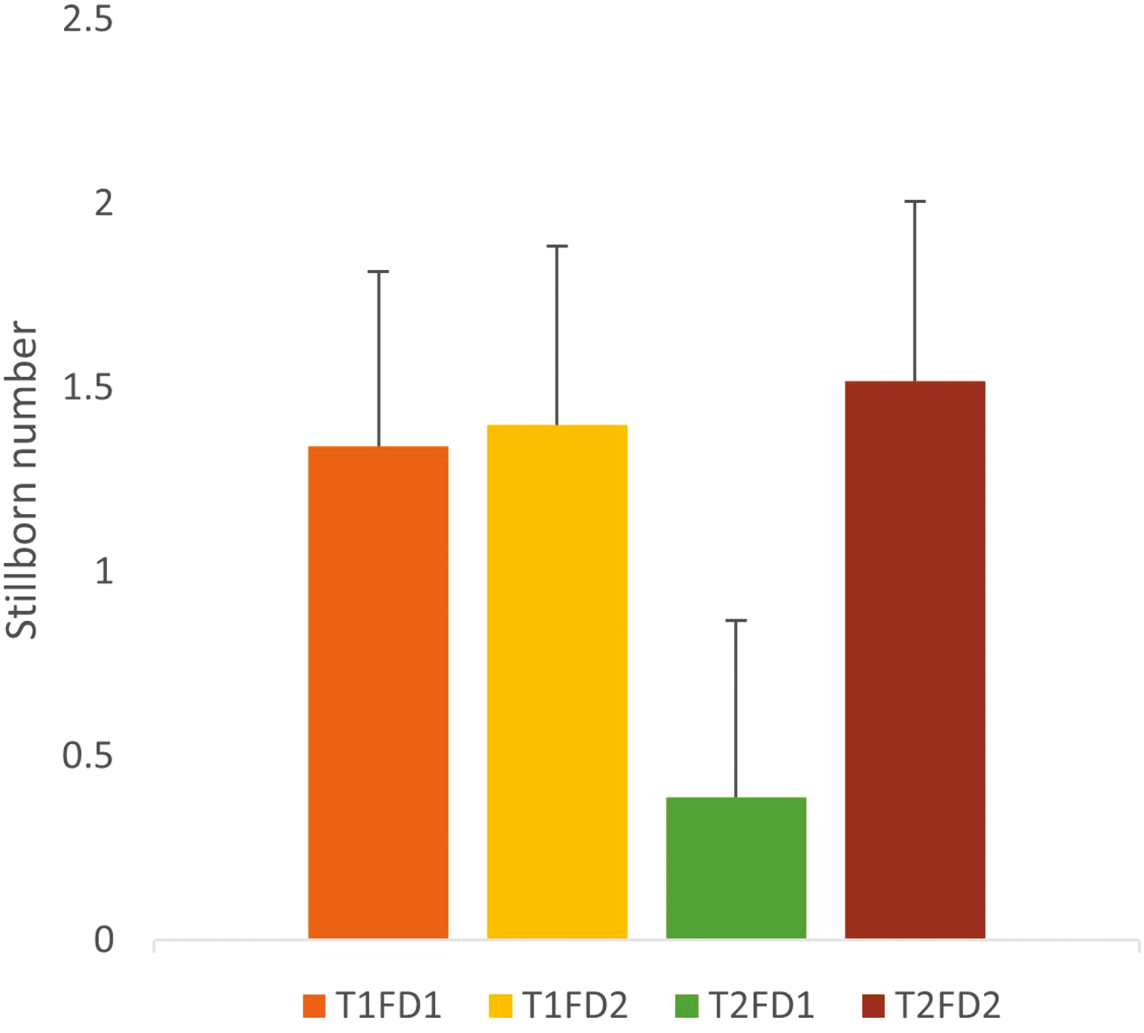
Mean ± standard error of stillborn number by the feeding treatment and farrowing duration for 33 sows bootstrapped at root of 8 (164 sow observations). Treatment 1 = one feed a day. Treatment 2 = two feeds a day. Farrowing duration 1, > 3.47 h. Farrowing duration 2, > 3.47 h. Accounting for sow backfat and room in the model.

Within the observed sows, the interaction of treatment and farrowing duration was shown to affect stillbirths (*P* < 0.001). Sows fed twice with shorter farrowing durations had fewer stillborn than those fed once with shorter farrowing duration (Figure 3). However, sows with longer farrowing durations did not differ in stillborn numbers regardless of feeding frequency.

### Lactation

Average daily gain was increased in piglets from sows fed once a day compared with those sows fed twice (Tables 2 and 3).

**Table 3.** Mean ± SE for average piglet lactation weight change by treatment, piglet birth weight, and sow entry backfat depth for all trial sows, 509 sow observations after bootstrapping

		N	Average piglet weight change
Treatment	1	292	4.98 ± 0.24
	2	217	4.80 ± 0.25
Piglet birthweight	1	122	4.87 ± 0.25^a^
	2	130	5.18 ± 0.25
	3	130	4.82 ± 0.25^ab^
	4	127	4.66 ± 0.25^b^

Treatment one, one feed a day. Treatment two, two feeds a day. Piglet birth weight (Wt) 1: <1.41 kg; Wt 2: 1.41 to 1.59 kg; Wt 3: 1.59 to 1.76 kg; Wt 4: > 1.76 kg.

Significance within piglet birth weight categories. Accounting for sow days from entry to farrowing, litter size, parity, sow backfat at entry, and room in the model.

Sows fed once a day lost more P2 backfat (0.56 ± 0.79 mm) than sows fed twice a day (0.02 ± 0.83; *P* = 0.03). However, the opposite was true for weight change, with twice-fed sows losing more weight to weaning than once-fed sows (1.31 ± 3.66 kg vs. 7.67 ± 3.73 kg; *P* < 0.001).

### Subsequent Reproduction

The wean to estrus period was not significantly different between single or twice-fed sows (9.97 ± 0.74 d and 9.23 ± 0.91 d; *P* = 0.23). As days from entry to farrow increased so did subsequent wean to estrus interval (5 d, 4.8 ± 1.04 d; 7 d, 10.5 ± 1.20 d; *P* < 0.001).

The percentage of sows culled from the herd, based on subsequent farrowing, was higher in those from once-fed than twice-fed populations (25.4% vs. 15.6%).

Subsequent total born was higher (*P* < 0.001) in twice-fed than once-fed sows (15.0 ± 0.50 vs. 16.3 ± 0.57), as was subsequent born alive (13.7 ± 0.34 vs. 14.4 ± 0.41; *P* = 0.001).

## DISCUSSION

The current study showed a biologically relevant reduction in farrowing duration when the transition sow feeding frequency was increased from one to two feeds a day, supporting the suggestion that the sows fed only once may have begun to suffer from an energy deficit thus extending their farrowing duration (Pedersen et al., 2016; Feyera et al., 2018). The presence of this difference regardless of a higher total born occurring in twice-fed sows further strengthens this argument as larger litters would be expected to increase the farrowing duration (van Dijk et al., 2005; Oliviero et al., 2010). Monitoring farrowing durations accurately both within and outside of normal staffed hours is a constraint on determining the effectiveness of management feeding strategies. Currently, few commercially available monitoring technologies exist for recording and reporting farrowing duration in production. Therefore, determining the benefit of increasing feeding frequency on an individual farm basis relies heavily on changes in stillborn numbers.

The stillborn number decreased in the subset of observed sows who received two feeds a day but not in the full dataset of sows. Sows in the full data set include sows that are farrowed outside of observation hours thus, while speculative, it is possible that the lack of supervision resulted in a failure to intervene in unobserved dystocia with a resultant increased stillbirth. It is also important to note that these sows started farrowing later in the day and thus there would have been a prolonged period between their last feed and onset of farrowing. Based on the subset of data and previous research (Feyera et al., 2018; Gourley et al., 2020; Ju et al., 2021), it is possible that these sows had longer farrowing durations and were associated with increased stillborn. Interestingly, when the interaction of treatment and farrowing duration is considered in the subset, it was seen that two feeds did decrease stillborn rate in shorter farrowing sows. However, there was a proportion of sows that maintained a higher farrowing duration and thus stillborn number regardless of feeding frequency. This suggests that there is a proportion of sows that can have improved farrowing performance and stillborn number through feeding frequency management but there are other factors influencing farrowing duration and the stillborn rate which are not affected by feeding frequency.

It is accepted that litter size, litter birth weight variation, and stillborn number all increase with increasing parity (Damgaard et al., 2003; Quesnel et al., 2008; Wientjes et al., 2012). Further, as litter size increases so does the total energy required by the sow to expel the piglets during farrowing (Oliveira et al., 2020). In contrast to previous studies, the mean litter size in our study was relatively small, suggesting that other factors may have influenced farrowing duration and/or the feed-to-farrow interval. It is well known that feeding strategies in previous lactations and gestation can impact lifetime performance, specifically sow condition and embryo quality (Bunter et al., 2006; Rozeboom, 2014). In the present study, although entry parity, backfat depth, and body weight were used to allocate sows to treatment, there was a large variation in backfat across the population. Feeding and management strategies are usually applied across the herd to maintain consistent conditions and performance outcomes. However, as seen in the current study, variation does occur both in the condition and performance of sows when a standard feeding strategy is used. Interestingly, Thongkhuy et al. (2020) suggested that backfat thickness at entry to the farrowing house does not impact farrowing duration but does influence stillborn number. The highest backfat score recorded prior to entry in their study was 24 mm which is at least 10 mm less than the highest recorded in the current study. Therefore, it is possible that sows with a higher backfat thickness at entry to the farrowing house could show a different effect on farrowing ease and output (Thongkhuy, 2020).

Backfat depth has also been shown to impact glucose tolerance in sows, with higher values resulting in lower glucose tolerance (Cheng et al., 2020). Although not investigated in this study, sows with increased backfat depths may have benefited more from increased feeding frequency, by enhancing glucose tolerance as a result of distributing the nutrient load across the day. Prior to and during farrowing, glucose is critical for uterine contractions and colostrum synthesis. However, once the dietary glucose has been metabolized, the sow must rely on stored energy to maintain her energy status (Serena et al., 2009; Feyera et al., 2018, 2019). This is consistent with the literature reporting that decreasing the time from last meal to the onset of farrowing can reduce the stillborn number (Feyera et al., 2018; Manu et al., 2019). This raises the suggestion that specific feeding regimes, such as increasing feeding frequency, could be used to tailor transition diets to the sow’s needs to optimize her farrowing and post-farrowing performance.

Sows fed once had higher average piglet gains and backfat loss than sows fed twice, while sows fed twice lost more weight. This would suggest that once-fed sows had better lactational performance than twice-fed. However, the piglet average weight gain was similar for both treatment groups by commercial standards (Brandt, 1998; Collins et al., 2017). Therefore, this difference is not outstanding or concerning. Surprisingly, more twice-fed sows were retained in the herd at subsequent mating and had a higher subsequent total born and born alive than once-fed sows. This suggests that although their current lactational performance was not as good as once-fed sows, their longevity was improved. As an economic benefit to the producer, this is of high value and could benefit long-term reproductive outputs. It should be acknowledged that there are many management and external factors which impact lactational performance and subsequent reproduction, such as previous and subsequent feeding regimes, and as such the sow transitional feeding management should be considered in context.

The present study focused on increasing feeding frequency to better distribute the energy available to the sow across the day under production conditions. Previous studies suggested that increasing feeding frequency to at least three times a day would result in a clearer difference in farrowing duration and lactation performance (Feyera et al., 2018; Manu et al., 2019). A different approach to increasing energy available would be comparing increased feeding frequency to increased feed allowance. This would explore the effects of different total feed intake prior to farrowing and the time between feed and farrow. Gourley et al. (2020) found that four smaller meals a day, compared with one large meal, increased piglet weight gain from 24 h but when sows were fed ad libitum prior to farrowing, weight gain was further improved. Although speculative, the lack of greater differences between one and two feeds a day in our study may be due to the feeding frequency not being increased enough, even though it would be impractical for many producers to accommodate more than this level of hand feeding. Further to this, anecdotal observations suggested that the sows receiving one feed often stood and vocalized when the second feeding occurred for the twice-fed sows. It could be suggested that the sows associated the stock person pushing a cart with receiving food and when not in receipt of food became distressed and/or restless. This increase in activity without receiving the energy from a second feed may have further been detrimental to energy reserves for farrowing (van Kempen, 2008; Feyera and Theil, 2017; Feyera et al., 2018). To minimize the impact of stock person movements and association with food, while increasing the ability of production to implement greater feeding frequencies, an automated feeding system would be recommended. However, as the benefits of increasing feeding frequency to reduce farrowing duration are still not in strong agreement in the literature, the authors hesitate to suggest this to producers until further research is conducted.

The sample size of the subset of sows for farrowing duration observations was small, due to the limitations of only being able to observe the sows in daylight hours which may limit the interpretation of these results. Despite this, the clear observation of a difference in farrowing duration suggests that more research into reducing the feed to farrowing interval is warranted. Although accounted for in all relevant statistical models, the occurrence of a higher total born in sows receiving two feeds a day may obscure the effectiveness of increasing feeding frequency in this experiment. This is a universal problem for all pre-farrow management experiments as an accurate prediction of total born piglets is practically impossible prior to farrowing under production conditions.

Increasing feeding frequency as a management tool is constrained by production capabilities. However, it could be useful for herds with larger litters or those prone to longer farrowings. Having two feeding sessions a day may provide an economic benefit (e.g., sow longevity and piglet numbers) to a producer if their sows exhibit a long farrowing duration and larger litter size. An alternative approach would be ad libitum feeding or automatic feeding systems capable of delivering multiple small feeds a day. Ad libitum would allow sows to self-satisfy their appetite and potentially benefit low backfat sows, but could increase the labor required to provide this (Cools et al., 2014). Automation would allow more control for later feeding sessions, specifically overnight, however, the cost of set-up and maintenance may be impractical for some producers and could also potentially reduce the observation and pick up of sow issues due to less monitoring.

## CONCLUSIONS

The outcomes of this study show that increasing feeding frequency may reduce the duration of farrowing, and potentially energy demand, but not the stillborn rate in a herd with short farrowing duration and smaller litter sizes. Feeding twice a day in late gestation did not improve sow body condition loss in lactation or reproductive output of sows compared with feeding once a day. Increasing feeding frequency should be considered for a system better designed to deliver feeding more accurately and automatically than does hand feeding. As litter sizes continue to increase, the applicability of research in transitional sow management and priority for production-appropriate outcomes is becoming more evident. Future research should prioritize effective monitoring technologies for accurate determination of the effects of sow transitional management changes on farrowing duration and stillborn numbers.
